# Supplementary material for: Cultryx: Precision Diagnostic Stewardship for Blood Cultures Using Machine Learning
Source: medRxiv. 2026 Mar 4:2026.02.27.26347214. Preprint. [Version 1] doi: 10.64898/2026.02.27.26347214 (PMC13004107; doi:10.64898/2026.02.27.26347214)
Supplement: Supplement 1 [file NIHPP2026.02.27.26347214v1-supplement-1.pdf]

medRxiv preprint doi: https://doi.org/10.64898/2026.02.27.26347214; this version posted March 4, 2026. The copyright holder for this preprint
(which was not certified by peer review) is the author/funder, who has granted medRxiv a license to display the preprint in perpetuity.
It is made available under a CC-BY 4.0 International license .

Supplementary Appendix A: Complete List of All Feature Definitions

Table 7: Final Feature Set and Definitions

**Feature**	**Category**	**Definition**
age	Demographics/Context	Patient age at encounter (years).
gender_binary	Demographics/Context	Binary sex indicator (as recorded in EHR).
bmi	Demographics/Context	Body mass index (kg/m^2).
has_any_line	Demographics/Context	Indicator for presence of any invasive line during encounter (binary).
hr_max	Vital Signs	Maximum heart rate (beats/min) during observation window.
sysbp_min	Vital Signs	Minimum systolic blood pressure (mmHg).
diabp_min	Vital Signs	Minimum diastolic blood pressure (mmHg).
resp_max	Vital Signs	Maximum respiratory rate (breaths/min).
spo2_min	Vital Signs	Minimum peripheral oxygen saturation (%).
temp_max_c	Vital Signs	Maximum body temperature (°C).
temp_delta_6h	Vital Signs	Maximum change in temperature over any 6-hour window (°C).
max_wbc	Laboratory	Maximum white blood cell count during window.
max_neutrophils	Laboratory	Maximum neutrophil percentage.
max_anc	Laboratory	Maximum absolute neutrophil count.
min_lymphocytes	Laboratory	Minimum lymphocyte percentage.
min_hgb	Laboratory	Minimum hemoglobin (g/dL).
min_plt	Laboratory	Minimum platelet count ($\times 10^3$/µL).
max_glucose	Laboratory	Maximum serum glucose (mg/dL).
max_lactate	Laboratory	Maximum serum lactate (mmol/L).
max_cr	Laboratory	Maximum creatinine (mg/dL).
max_crp	Laboratory	Maximum C-reactive protein (mg/dL).
shock_index	Derived	Ratio of maximum heart rate to minimum systolic blood pressure (shock_index = hr_max / sysbp_min).
fever_flag	Derived	1 if temp_max_c $\geq$ 38.0°C, else 0.
hypotension_flag	Derived	1 if sysbp_min $\leq$ 90 mmHg, else 0.
tachycardia_flag	Derived	1 if hr_max $\geq$ 100 beats/min, else 0.
tachypnea_flag	Derived	1 if resp_max $\geq$ 22 breaths/min, else 0.
hypoxia_flag	Derived	1 if spo2_min $\leq$ 94%, else 0.
hyperglycemia_flag	Derived	1 if max_glucose $>$ 127 mg/dL, else 0.
hyperlactate_flag	Derived	1 if max_lactate $>$ 2.0 mmol/L, else 0.
renal_impairment_flag	Derived	1 if max_cr $>$ 1.2 mg/dL, else 0.
inflammatory_flag	Derived	1 if max_crp $>$ 10 mg/dL, else 0.
anemia_flag	Derived	1 if min_hgb $<$ 12 g/dL, else 0.
thrombocytopenia_flag	Derived	1 if min_plt $<$ 150 $\times 10^3$/µL, else 0.
neutrophilia_flag	Derived	1 if max_neutrophils $>$ 70%, else 0.
lymphopenia_flag	Derived	1 if min_lymphocytes $<$ 20%, else 0.
composite_instability_flag	Derived	Set to 1 if any of fever_flag, hypotension_flag, or hyperlactate_flag are present; otherwise 0.

medRxiv preprint doi: https://doi.org/10.64898/2026.02.27.26347214; this version posted March 4, 2026. The copyright holder for this preprint (which was not certified by peer review) is the author/funder, who has granted medRxiv a license to display the preprint in perpetuity.
It is made available under a CC-BY 4.0 International license .

Supplementary Appendix B: Full Prompt for Generative AI (GPT-5) Application of the Fabre Framework

Full GPT-5 Prompt

// Role
You are an infectious diseases physician tasked with classifying an adult patient's risk of bacteremia at the time of a clinical event or blood culture draw, using criteria from Fabre et al., "Does This Patient Need Blood Cultures? A Scoping Review of Indications for Blood Cultures in Adult Nonneutropenic Inpatients" (DOI: https://doi.org/10.1093/cid/ciaa039).

// Procedure Checklist
Begin with a concise checklist (3-7 bullets) of what you will do; keep items conceptual, not implementation-level.
- Review provided note and EHR data.
- Determine if patient meets exclusion criteria.
- Sequentially check against each risk tier (High, Intermediate, Low) as defined.
- Assign the highest triggered tier or "Undetermined" as appropriate.
- Extract 1-3 verbatim supporting quotes.
- Assess and assign confidence score per guideline.
- Compose and validate concise rationale aligning with Fabre categories/examples.

// Context and Criteria
Scope and Assumptions (strictly enforced):
- Population: Adults (greater than or equal to 18 years), non-neutropenic patients. If data indicate pediatric status or ANC less than 500/µL, return "Undetermined".
- Use only information present in the note and structured EHR data provided. Do not create or assume data.
- If multiple conditions apply, assign the highest applicable risk tier.

Fabre-Based Risk Tier Definitions (category logic and examples maintained):
- HIGH RISK: Signs of severe sepsis or septic shock, or conditions predisposing to endovascular infection. Examples: catheter-associated bloodstream infection, discitis/native vertebral osteomyelitis, epidural abscess, meningitis, non-traumatic native septic arthritis, ventriculo-arterial shunt infection.
- INTERMEDIATE RISK: Symptoms of systemic infection or localized infections with systemic potential, but without overt severe sepsis. Examples: acute pyelonephritis, cholangitis, non-vascular shunt infections, prosthetic vertebral osteomyelitis, severe community-acquired pneumonia (PSI IV–V). Low-to-intermediate: cellulitis with significant comorbidities, ventilator-associated pneumonia.
- LOW RISK: Non-bacterial syndromes or absence of significant systemic infection signs. Examples: isolated fever/leukocytosis, non-severe cellulitis, lower UTI (cystitis, prostatitis), non-severe community-acquired pneumonia, health-care–associated pneumonia, post-op fever within 48 hrs of surgery.
- Severity interpretation: Use explicit mentions of "severe sepsis," "septic shock," vasopressor use, or acute end-organ dysfunction linked to infection. PSI class IV–V corresponds to severe CAP. Do not escalate risk tier without explicit severity cues.

// Decision Algorithm
1. Exclude Out-of-Scope: If pediatric (age ¡18) or neutropenic, classify as "Undetermined".
2. Check for HIGH RISK: Severe sepsis/septic shock or endovascular-predisposing condition (listed examples or clear equivalents).
3. If not, check INTERMEDIATE RISK: Systemic infection symptoms or localized infection with

medRxiv preprint doi: https://doi.org/10.64898/2026.02.27.26347214; this version posted March 4, 2026. The copyright holder for this preprint (which was not certified by peer review) is the author/funder, who has granted medRxiv a license to display the preprint in perpetuity.
It is made available under a CC-BY 4.0 International license .

systemic risk (examples), without severe sepsis.
4. If not, check LOW RISK: Use provided examples and confirm absence of systemic signs.
5. If insufficient or contradictory information, classify as "Undetermined".
6. If multiple tiers are triggered, assign the highest tier.

After classification, double-check that (a) the highest applicable tier is selected, (b) verbatim quotes support the assigned tier, and (c) rationale accurately references Fabre categories/examples.

// Output Format (strict JSON only)
"Classification": "HIGH" — "INTERMEDIATE" — "LOW" — "Undetermined", "Confidence": number, "Verbatim": ["quote1", "quote2", "quote3"], "Rationale": "2–4 sentences explaining how the evidence matches the tier, referencing Fabre categories/examples."

// Output Constraints
- Output strictly as valid JSON. No preamble, markdown, or extra keys.
- Verbatim quotes must be exact excerpts from the input text (180 characters each).
- Confidence scoring: 0.9–1.0 (explicit qualifiers and clear example matches), 0.6–0.8 (probable with partial data), 0.3–0.5 (ambiguous, weak cues), 0.0–0.2 (insufficient or contradicts—likely "Undetermined").

// Inputs Provided
- Unstructured note text (emergency department provider note).
- Optional structured EHR data (vitals, labs, problem list, etc.).

// Edge Cases Quality Checks
Quality Control Before Submission:
- Ensure classification matches the highest triggered tier.
- Ensure verbatim quotes directly support the assigned tier.
- Ensure rationale is concise and aligns with Fabre categories/examples.
- Output only valid JSON as described above.
